# *Pax3* Hypomorphs Reveal Hidden Pax7 Functional Genetic Compensation *in Utero*

**DOI:** 10.3390/jdb10020019

**Published:** 2022-05-17

**Authors:** Hong-Ming Zhou, Simon J. Conway

**Affiliations:** 1 Department of Dermatology, Indiana University School of Medicine, Indianapolis, IN 46202, USA; zhouhm00@yahoo.com; 2 Herman B. Wells Center for Pediatric Research, Indiana University School of Medicine, Indianapolis, IN 46033, USA

**Keywords:** Pax3 and Pax7, functional genetic compensation, cardiac neural crest, heart defects, craniorachischisis, mouse embryo, hypomorph, lineage mapping, genetic cell ablation

## Abstract

Pax3 and Pax7 transcription factors are paralogs within the *Pax* gene family that that are expressed in early embryos in partially overlapping expression domains and have distinct functions. Significantly, mammalian development is largely unaffected by *Pax7* systemic deletion but systemic *Pax3* deletion results in defects in neural tube closure, neural crest emigration, cardiac outflow tract septation, muscle hypoplasia and *in utero* lethality by E14. However, we previously demonstrated that *Pax3* hypomorphs expressing only 20% functional Pax3 protein levels exhibit normal neural tube and heart development, but myogenesis is selectively impaired. To determine why only some Pax3-expressing cell lineages are affected and to further titrate Pax3 threshold levels required for neural tube and heart development, we generated hypomorphs containing both a hypomorphic and a null *Pax3* allele. This resulted in mutants only expressing 10% functional Pax3 protein with exacerbated neural tube, neural crest and muscle defects, but still a normal heart. To examine why the cardiac neural crest appears resistant to very low Pax3 levels, we examined its paralog *Pax7*. Significantly, Pax7 expression is both ectopically expressed in Pax3-expressing dorsal neural tube cells and is also upregulated in the Pax3-expressing lineages. To test whether this compensatory Pax7 expression is functional, we deleted *Pax7* both systemically and lineage-specifically in hypomorphs expressing only 10% Pax3. Removal of one *Pax7* allele resulted in partial outflow tract defects, and complete loss of *Pax7* resulted in full penetrance outflow tract defects and *in utero* lethality. Moreover, combinatorial loss of Pax3 and Pax7 resulted in severe craniofacial defects and a total block of neural crest cell emigration from the neural tube. *Pax7^Cre^* lineage mapping revealed ectopic labeling of Pax3-derived neural crest tissues and within the outflow tract of the heart, experimentally confirming the observation of ectopic activation of Pax7 in 10% *Pax3* hypomorphs. Finally, genetic cell ablation of *Pax7^Cr^*^e^-marked cells is sufficient to cause outflow tract defects in hypomorphs expressing only 10% Pax3, confirming that ectopic and induced Pax7 can play an overlapping functional genetic compensational role in both cardiac neural crest lineage and during craniofacial development, which is normally masked by the dominant role of Pax3.

## 1. Introduction

The nine transcription factors of the *Paired box* (*Pax*) gene family have all been shown to play various vital roles in lineage specification during embryonic development but can also persist in adult stem and/or progenitor populations [1,2,3,4,5]. Moreover, *Pax* genes are highly conserved and contain a bipartite DNA binding paired domain and are often associated with a homeodomain, which itself is able to form both homo- and hetero-dimers on DNA [6]. Additionally, *Pax* genes exhibit an unusual gene dosage requirement, as both loss-of-function heterozygous mutations and gain-of-function over-expresser transgenic mice often cause phenotypically similar semi-dominant phenotypes [2]. Thus, despite the presence of many transgenic and mutant mice lines, the complexity of multiple family members, separate regulatory capabilities and gene dosage effects, it still remains mostly unclear how *Pax* mutations differentially affect tissues within such a wide range of cell types.

*Pax3* and *Pax7* are a subgroup of the *Pax* gene family that arose via gene duplication and are the most structurally and functionally related Pax members [7,8]. In early mouse embryonic development, paralogous Pax3 and Pax7 are both expressed in distinct and overlapping domains of the central nervous system and the myogenic precursors of skeletal muscle [9,10]. However, *Pax3* is initially expressed at neural plate stages and precedes that of *Pax7*, which starts after neural tube closure [11]. Consequently, while *Pax3* loss of function (*Sp^2H^* and *Pax3^Δ5^* alleles) leads to a wide spectrum of neural tube, neural crest and muscle congenital defects and *in utero* lethality by E14 [12,13], mouse development is largely unaffected by *Pax7* loss-of-function mutation [11]. Specifically, *Pax3* null mice have demonstrated that Pax3 plays a critical role in neural crest lineages that migrate from the dorsal neural tube, including cardiac neural crest cells that invade the arterial pole of the developing heart and are required for outflow tract septation and *in utero* survival [2,12,13,14,15]. Moreover, we demonstrated that lineage-restricted deletion of mouse Pax3 using the *Pax3^flox^* allele from only the dorsal-most Pax3-expression domain in early pre-migratory neural crest within the neural tube (normally a Pax7-free domain) does not affect the cardiac neural crest derivative and this is what underlies Pax3’s requirement during cardiac neural crest and embryonic outflow tract septation [13]. Further, using a hypermorphic *Pax3* (*Pax3^neo^*) allele, we showed a lineage-specific response to ~80% loss of Pax3 protein expression, with myogenesis of the limb and tongue being most sensitive to reduced Pax3 levels, but the neural crest-derived lineages and neural tube were all unaffected [2]. Intriguingly, elevated levels of paralogous Pax7 protein were present in unaffected *Pax3^neo^* hypermorphic neural tube and epaxial somatic components [2]. Similarly, *Pax7* mRNA and protein are both strongly upregulated and expanded dorsally into the Pax3-only expression domain in *Pax3* null (*Sp* allele) embryonic neural tube and somites, suggesting that Pax3 may normally function to repress Pax7 [16]. However, despite Pax7 being one of the earliest markers of avian neural crest [17] and *Pax7^Cre^/R26^YFP^* lineage mapping showing some mammalian neural crest contribution [18], Pax7 is not thought to play a major functional role in mouse heart morphogenesis [11]. Interestingly, *Pax3 (Pax3^Δ5^)/Pax7 (Pax7^Δ2^)* double heterozygotes exhibit a gain-of-function phenotype, as 40% of double heterozygotes develop postnatal hydrocephalus, which is not seen in either single heterozygote [19]. Significantly, *Pax3 (Sp^2H^)/Pax7* (*Pax7^Δ1^*) double null embryos die by E11 and exhibit exacerbated neural tube deformities [20]. Given that Pax3 deficiency alone is sufficient to cause cardiac neural crest-associated defects, it was not possible to examine the additive impact of Pax7 loss upon heart development in *Pax3/7* double nulls. Nevertheless, as *Pax3/7* double null lethality is earlier and neural tube defects are more severe than just *Pax3* nulls (which die around E14), this suggests these two Pax genes may share redundant functions. To test Pax3/7 redundancy and functional genetic compensation, the *Pax7* coding region was knocked-in into the *Pax3* locus (*Pax3^Pax7-ILZ/Pax7-ILZ^* allele), demonstrating that Pax7 can substitute for Pax3 function within dorsal neural tube/neural crest cell lineages, and during somite development, but not in long-range migration of muscle progenitor cells [21]. This elegant replacement experiment established that in the absence of Pax3, one copy of the structurally related Pax7 can functionally compensate for loss of both *Pax3* alleles when ectopically expressed within the dorsal-most neural tube and somite Pax3-derived tissues. However, as knock-in *Pax7* is now expressed at sites where Pax3, but not Pax7, is normally expressed and at an earlier stage of development, combined with existing endogenous Pax7 expression in overlapping Pax7-derived tissues, along with the findings that Pax3 may negatively regulate Pax7 [2,16,22,23]; this approach was not able to directly test Pax3/7 interplay, individual dosage requirements, or whether there is any functional genetic compensation via the upregulation of compensatory Pax7 within Pax3-derived lineages. Additionally, it also remains unclear why in *Pax3* the loss of function nulls, why not all neural crest derivatives are equally affected in the absence of Pax3, and why some lineages appear to be more sensitive at the expense of others. For instance, cardiac, enteric, melanocyte-derived and Schwann cell precursor neural crest cells are all affected by absence of Pax3, but the cranial neural crest appears unaffected [24,25].

Using four separate yet interdependent approaches, we took advantage of our novel *Pax3^neo^* hypomorphic allele, which encodes a reduced Pax3 level (20% in *Pax3^neo/neo^*) but does not exhibit any cardiovascular defects or survive until birth [2]. Firstly, we combined our *Pax3^neo^* hypermorphic and *Pax3* loss-of-function *Pax3^Δ5^* null alleles to further reduce Pax3 expression. Second, to test whether cardiac neural crest morphogenesis resistance to a 90% reduction in Pax3 levels in *Pax3^neo^/^Δ^^5^* embryos is attributed to a Pax7 redundant role or functional genetic compensation, we incorporated a *Pax7* null allele (*Pax7^Δ2^*). Third, we generated *Pax3/7* double mutants on a defined genetic background that live up to E14 and exhibit exacerbated failed closure along the entire neural tube, severe facial morphogenesis defects, fully penetrant outflow tract defects and a complete lack of Pax3-derived neural crest emigration to the embryonic heart. Fourth, we used a *Pax7^Cre^* allele to both lineage map and genetically ablate Pax7-expressing cells with both wildtype and hypomorphic backgrounds. Despite endogenous Pax7 never having been implicated directly in the formation of the heart, genetic cell ablation of *Pax7^Cr^*^e^ lineage in *Pax3^neo^/^Δ^^5^* mutants result in cardiac outflow tract defects, revealing an unappreciated cardiovascular capability for Pax7. Collectively, these data demonstrate that ectopic Pax7-expressing neural crest cells can compensate for reduced Pax3 during *Pax3* hypermorphic neural tube closure, craniofacial development, neural crest emigration and outflow tract morphogenesis and that ablation of the ectopic *Pax7^Cre^*-expressing cell lineage is sufficient to cause congenital heart defects in *Pax3* hypomorphic mammals. Here, we show that structurally related wildtype Pax3 and Pax7 proteins are also functionally transposable.

## 2. Materials and Methods

### 2.1. Genetically Modified Mice

The generation of *Pax3* (MGI:97487) conditionally targeted mice, in which exon 5 was flanked with loxP sites [26] and the generation of heterozygous *Pax3* mice (*Pax3^Δ5^*^/*+*^) on C57BL/6 backgrounds via germline deletion of exon 5 was described previously [13]. Moreover, the hypermorphic effect of the intact neomycin-containing *Pax3* allele (*Pax3^neo^*) has also been reported [2]. *Pax7* (MGI:97491) mutant allele (*Pax7^Δ2^*) was generated by germline deletion of exon2 [27] and backcrossed onto a *C57Bl/6* background. *In utero* embryos and fetuses with different combinations of *Pax3* and *Pax7* allelic status were collected and genotyped as described [2,13,27]. For lineage-mapping experiments, compound *Pax3^∆5/+^* and *Pax3^neo/+^* mice were bred onto a homozygous *R26r^LacZ^* indicator background and intercrossed with either *Wnt1^Cre^* or *Pax7^Cre^* mice [28,29]. Significantly, the *Pax7^Cre^* knock-in Cre cassette was placed within the 3′ UTR of *Pax7* gene locus, enabling the modified allele to recapitulate wildtype Pax7 reporter expression while maintaining wildtype Pax3 and Pax7 protein levels (Supplemental Figure S1). *Pax7^Cre^*-mediated genetic cell ablation was carried out using *R26^-eGFP-DTA^* (*R26r^DTA^* [30]) mice as described [31]. *Cre, LacZ* or *R26r^DTA^* transgenes were detected via PCR genotyping as described [13]. Animal procedures and experimental conditions were refined to minimize harm to animals and performed with the approval of the Institutional Animal Care and Use Committee of Indiana University School of Medicine (protocol #20101). For timed pregnancies, the day of observed vaginal plug was designated embryonic day 0.5 (E0.5).

### 2.2. Histologic Analysis

Immunohistochemical and X-gal Staining: Isolation of tissues, fixation, processing, and whole-mount staining for β-galactosidase was performed as described [13,31]. Immunostaining was carried out using ABC kit (Vectorstain, Burlingame, CA, USA) with DAB and hydrogen peroxide as chromogens, as described [2]. The dilution of primary antibodies was 1:200 for goat anti-Pax3 (Santa Cruz sc-7748); 1:200 for mouse anti-Pax7 (Hybridoma Bank, Iowa, IA, USA); 1:5000 for mouse anti-alpha smooth muscle actin (αSMA; Sigma); and 1:48,000 for goat anti-neuron specific-β3 Tubulin (NSβT) (Abcam, Cambridge, UK). Antibody diluent (Vectorstain, Burlington, CA, USA), without primary antibodies, was used for negative controls. For each assay, whole embryos and/or serial sections were examined for at least three individual embryos of each genotype. A specific signal was only noted in at least three consecutive serial sections at each developmental stage. Duplicate wildtype littermates were used as age-matched controls.

### 2.3. Western Blot Analysis

For Western blot analysis, individual E9.5 and 10.5 embryos (*n* = 3–5 of each genotype) were homogenized in protein lysis buffer and resolved using 10% SDS-PAGE (Bio-Rad, Hercules, CA, USA), transferred to nitrocellulose and blocked for 1 h, as described [2]. Blots were probed with mouse monoclonal anti-Pax3 (1:2000 dilution) or monoclonal anti-Pax7 (1:2000 dilution) antibodies (both obtained from the Hybridoma Bank) in blocking solution. The signal was detected via ECL^Plus^ (Amersham, UK) with peroxidase-conjugated goat anti-mouse secondary antibody (1:5000 dilution, Promega, Madison, WI, USA). To verify equal loading, all blots were subsequently stripped (0.2 M NaOH for 5 min at room temperature), washed, re-blocked and then probed with loading control mouse anti-Actin antibody (1:000 dilution, Sigma, Burlingame, MA, USA). X-ray films were scanned, and signal intensity was measured using ImageJ software (downloaded from wsr@nih.gov accessed on 1 February 2022).

### 2.4. Reverse Transcriptase-PCR Analysis

Total RNA was isolated from genotyped embryos individually using Trizol (Invitrogen, Waltham, MA, USA) following manufacturer’s recommendations. cDNA was synthesized in a 20 uL reaction with 1 ug of RNA using random primers (SuperScrip^TM^ III First-Strand Synthesis System for RT-PCR, Invitrogen, Waltham, MA, USA). cDNAs were amplified in duplicate with specific primers (Table 1) and repeated with at least 3 separate isolates and normalized using *GAPDH* loading control as described [2,19]. Amplified PCR bands were cloned and sequenced to verify identify. Densitometry was quantified from at least 3 samples and the combined data graphically displayed. Differences were considered to be statistically significant for those with *p* < 0.05. Statistical analysis was performed with Prism software version 5.02 (GraphPad Software, San Diego, CA, USA).

## 3. Results

### 3.1. Select Neural Crest Lineages Are Resistant to a Drop in Pax3 Levels

**Figure 1:**
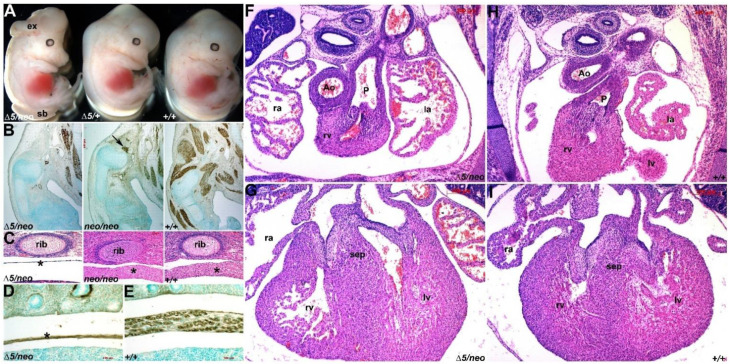
*Pax3^∆5/neo^* hypomorphic mutants do not exhibit any CHDs. (**A**) Whole fetal views of E14 *Pax3^∆5/neo^*, *Pax3^∆5/+^* and wildtype (+/+) littermates, reveals presence of spina bifida (sb) and exencephaly (ex) neural tube defects in only *Pax3^∆5/neo^* mutants. (**B**) Immunohistochemistry using αSmooth muscle actin (brown) at shoulder level in three fetuses depicted in (**A**), detects the muscle lineage. Note in a gene dosage-responsive style, there is a complete absence of limb musculature in *Pax3^∆5/neo^*, and a severe reduction in *Pax3^∆5/+^* compared to normal musculature in the wildtype littermate. Fibers of the triceps brachii (arrow in middle panel) are still present but the extensor and flexor muscles of the forelimb are missing in *Pax3^∆5/+^* embryos. (**C**) (H,E) staining of the diaphragm at E17.5. While the thickness of diaphragm is similar in *Pax3^neo/neo^* and +/+, further reduction in Pax3 resulted in hypoplastic *Pax3^∆5/neo^* diaphragm (*). (**D**,**E**) Immunohistochemistry using αSmooth muscle actin revealed robust musculature in both wildtype diaphragms, but a lack of myofiber arrangement in *Pax3^∆5/neo^* mutants. (**F**–**I**) (H,E) analysis of E15.5 hearts at both outflow tract and interventricular septum (sep) levels revealed that *Pax3^∆5/neo^* mutant hearts are grossly unaffected by the ~90% reduction in Pax3 levels. Both the wildtype (**H**,**I**) and *Pax3^∆5/neo^* (**F**,**G**) hearts exhibit separate aorta (Ao) and pulmonary trunks (P) and normal ventricular septation, as well as similar normal valvular structures. Abbreviations: ra, right atria; la, left atria; rv, right ventricle; lv, left ventricle. Scale (**B**), 500 μm; (**C**–**E**), 100 μm; (**F**–**I**), 200 μm.

Using our hypomorphic *Pax3^neo^* allele, we previously demonstrated that ~20% Pax3 protein levels are sufficient to initiate/maintain all non-myogenic biological processes. Significantly, *Pax3^neo/neo^* mutants have hypoplastic muscle defects but do not exhibit neural tube, neural crest and/or cardiovascular defects and thus survive until birth [2]. To further titrate the Pax3 protein threshold to enable us to examine Pax3 dosage requirements within the various Pax3-expressing cell lineages, we combined our *Pax3^neo^* hypermorphic and Pax3 loss-of-function *Pax3^Δ5^* null alleles. As expected, given 100% *Pax3^neo/neo^* lethality [2], no *Pax3^neo/∆5^* offspring survived neonatally (*n* = 7 litters). However, 100% of E19 *Pax3^neo/∆5^* mutants were present at Mendelian ratios (*n* = 31 *Pax3^neo/∆5^* (~24.5%) from 125 total offspring examined, suggesting they all survive until birth. Considering that dams often destroy newborns if they are unhealthy right after delivery, we collected E19 fetuses to observe their respiratory behavior. Both wildtype and *Pax3^neo/∆5^* mutants (*n* = 5 of each genotype) exhibited the typical purple-to-pink skin color change seen after taking their first breath, but *Pax3^neo/∆5^* mutants quickly became cyanotic (data not shown), indicating respiratory failure as a cause of death. Consistent with an additional loss of Pax3 and given the known Pax3 expression domains [9], *Pax3^neo/∆5^* fetuses now exhibit phenotypes that are more consistent with documented *Pax3* null phenotypes [12]. Strikingly, while the frequency of neural tube defects in *Pax3^Δ5/+^* (1% spina bifida, *n* = 72) and *Pax3^neo/neo^* (2% spina bifida, *n* = 200; a rate close to spontaneous neural tube defects in our mouse colony) mutants is low, most *Pax3^neo/∆5^* mutants (66%) now have neural tube defects (43% exhibit exencephaly with/without spina bifida; 23% spina bifida only, *n* = 100; Figure 1A). In addition to increased frequency of neural tube defects, *Pax3^neo/∆5^* mutants more readily feature dysmorphic forelimbs due to exacerbated muscular absences (Figure 1B). Additionally, *Pax3^neo/∆5^* mutants exhibit severe diaphragmatic muscle hypoplasia that was not observed in *Pax3^neo/neo^* mutants (Figure 1C,D), and this is most likely the primary cause of the observed perinatal respiratory failure. To examine whether compromised neural crest and/or heart morphogenesis may also contribute to lethality, we histologically examined the *in utero* E14 cardiovascular system. Serial section analysis failed to identify any characteristic *Pax3* null defects [12] such as persistent truncus arteriosus (PTA), double outlet right ventricle (DORV) and/or interventricular defects (VSDs) and congenital heart defects (Figure 1F–I). Nevertheless, as the neural crest gives rise to many tissues, including the dorsal root ganglia (DRG) and thymus, both of which are hypoplastic in various *Pax3* mutants [12,32], we also examined these. Despite the lack of any cardiac neural crest-associated outflow tract defects, *Pax3^neo/∆5^* mutant DRGs and thymus are reduced in size (not shown), indicating that only select neural crest lineages are compromised via further reduction in Pax3. Thus, despite the exacerbated muscular, neural crest and neural tube defects, the observed normal septation of outflow tract in *Pax3^neo/∆5^* mutants suggests that the cardiac neural crest lineage is the most resistant to Pax3 reductions.

**Figure 2:**
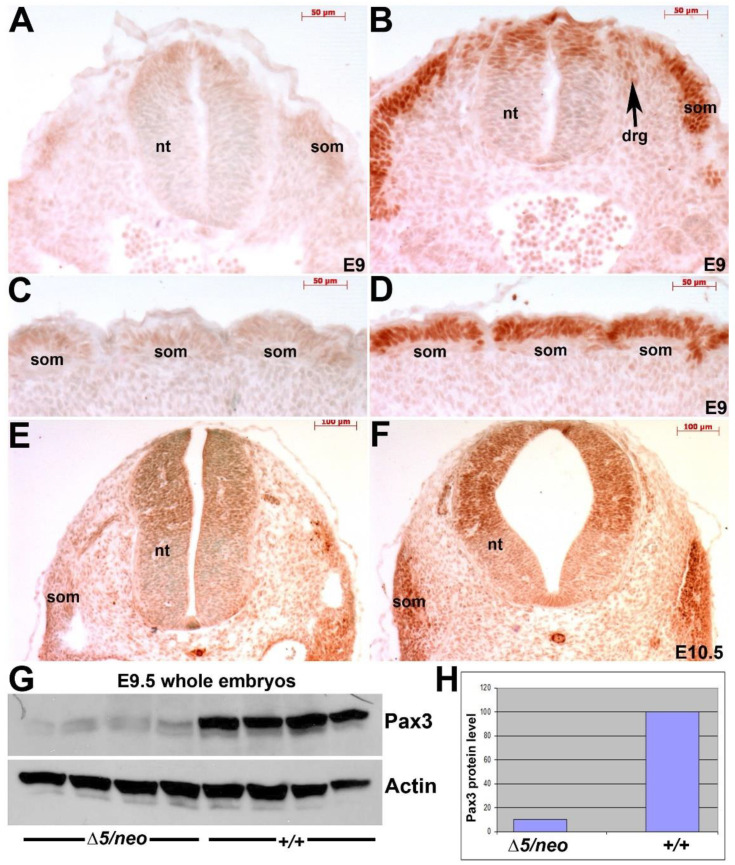
Pax3 protein expression is globally reduced ~90% in *Pax3^Δ5/neo^* hypomorphic mutants. (**A**–**D**) IHC reveals reduced Pax3 protein levels in E9.5 *Pax3^∆5/neo^* mutants (**A**,**C**) compared to robust expression in age-matched littermate control embryo (**B**,**D**) within the dorsal neural tube (nt), dorsal root ganglia (DRG) and somites (som). Moreover, Pax3 protein is suppressed in both anterior (**A**,**B**) and caudal (**C**,**D**) somites. (**E**,**F**) Pax3 protein levels remain reduced throughout the entire E10.5 *Pax3^∆5/neo^* mutant embryo (**E**) compared to wildtype littermates (**F**). (**G**,**H**) Western analysis of Pax3 in E9.5 whole embryo lysates (*n* = 4 individuals of each genotype) revealed a significant reduction in full-length Pax3 levels, as band size identical to wildtype. Pax3 level were determined by densitometry and normalized to Actin expression (**H**). Relative to 100% expression in wildtype, there is only 10% Pax3 within the *Pax3^∆5/neo^* mutants. Scale (**A**–**D**), 50 μm; (**E**,**F**), 100 μm.

To determine the extent of the hypomorphic effect and confirm that *Pax3^neo/∆5^* mutants exhibit a further reduction in Pax3, we examined Pax3 expression. Immunohistochemistry (IHC) revealed reduced Pax3 protein in all *Pax3*-expressing tissues examined, including E9.5 and 10.5 dorsal neural tube and somites (Figure 2A–F). Consistent with IHC, Western analysis and densitometry confirmed E9.5 *Pax3^neo/∆5^* hypomorphs express full-length Pax3 protein but at only ~10% Pax3 levels relative to 100% in wildtype littermates (Figure 2G,H). Similarly, RT-PCR analysis of *Pax3* mRNA using primers that amplify both the 5′ and 3′ *Pax3* transcripts, respectively, yielded similar amplicon expression levels in *Pax3^neo/∆5^* and *Pax3^+/+^* embryos, indicating that all three *Pax3* alleles (*Δ5, neo, wildtype*) were transcribed equally (see Supplemental Figure S1). However, RT-PCR designed to amplify the exon5-exon6 region (cannot amplify *Pax3* transcribed from *Pax3^∆5^* allele as *exon5* deleted) confirmed a ~90% instead of 50% reduction in amplification within E9.5 *Pax3^neo/∆5^* embryos, suggesting the message transcribed from *Pax3^neo^* allele undergoes impaired splicing due to the presence of intervening neomycin cassette inserted between exon5 and exon6. In agreement with this suggestion, RT-PCR with prolonged elongation time yielded an extra larger band exclusively in *Pax3^neo/∆5^* samples (*n* = 3). Sequencing of the extra band confirmed they are neomycin-containing fragments. Thus, RT-PCR analysis using complimentary amplification strategies demonstrates that the *Pax3^neo^* allele is transcribed with equal efficiency, but the resulting mRNA cannot undergo normal exon5-exon6 splicing, leading to reduced levels of normal mature mRNA to encode wildtype Pax3 protein (see Supplemental Figure S1). This confirmed that the ~90% loss of Pax3 protein in *Pax3^neo/∆5^* mutants is a consequence of a combination of the loss of a *Pax3* allele and impaired splicing of mRNA transcribed from the *neo* allele. The affected mRNA splicing efficiency thus differentiates *Pax3^neo/∆5^* from *Pax3^+/∆5^*, which expresses ~50% Pax3 protein and develops largely unaffected.

### 3.2. Reduction in Pax3 Expression Elicits Ectopic and Elevated Pax7 Expression

**Figure 3:**
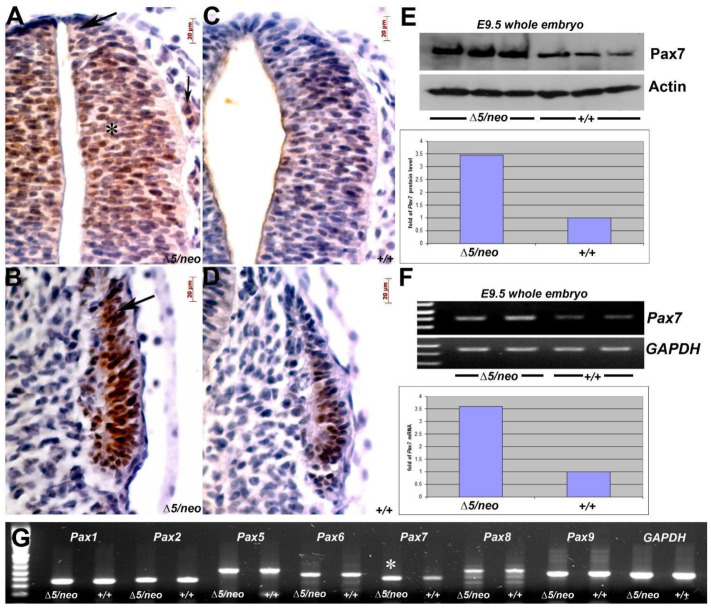
Reduced Pax3 in *Pax3^∆5/neo^* mutants elicits ectopic and elevated Pax7 expression. (**A**–**D**) Pax7 IHC in E10.5 *Pax3^∆5/neo^* and wildtype embryos revealed that Pax7 expression is both upregulated (indicated by bold arrow in **A**) and dorsally expanded (indicated by * in **A**) into the most dorsal region that is usually devoid of Pax7 with the *Pax3^∆5/neo^* neural tube (**A**) compared to wildtype littermate (**C**). Additionally, ectopic Pax7 is also observed in migrating *Pax3^∆5/neo^* neural crest cells adjacent to the dorsal neural tube (small arrow in **A**) but not wildtype (**C**). Furthermore, Pax7 is upregulated and dorso-laterally expanded in the *Pax3^∆5/neo^* somites (arrow in **B**) compared to wildtype (**D**). (**E**) Western blot verification of Pax7 upregulation in *Pax3^∆5/neo^* whole embryo lysate at E9.5. Three individuals for each genotype were analyzed and densitometry reveals a ~3.4 fold increase in Pax7 protein compared to Actin levels. (**F**) RT-PCR analysis of *Pax7* mRNA levels in duplicate wildtype and *Pax3^∆5/neo^* E9.5 in whole embryos. Densitometry confirms a ~3.5x fold increase in *Pax7* compared to *GAPDH* levels in *Pax3^∆5/neo^* mutants. (**G**) Comparative RT-PCR analysis of Pax gene family in *Pax3^∆5/neo^* mutants. Note, only *Pax7* mRNA was elevated (indicated by *) in *Pax3^∆5/neo^* embryos when Pax3 is reduced, as *Pax1, 2, 5, 6, 8* and *9* mRNA levels were similar to wildtype when normalized to GAPDH levels. Note *Pax4* mRNA is restricted to the pancreas and was not detectable in both genotypes (not shown) under the given conditions. Scale (**A**–**D**), 20 μm.

As shown, *Pax3^neo/neo^* hypomorphs can upregulate structurally related Pax7 [2], Pax3 may inhibit Pax7 [22,23] and *Pax3/7* double heterozygotes and double nulls each exhibit additive phenotypic severity [16,19], so we investigated whether a further reduction in Pax3 induces supplemental Pax7. As expected, IHC confirmed that further reduction in Pax3 results in elevated Pax7 expression in all Pax7-expression domains within both the E10.5 *Pax3^neo/∆5^* neural tube and somites. However, there was also an ectopic expansion of the Pax7 expression domains into the *Pax3^neo/∆5^* dorsal-most neural tube and the emigrating neural crest (Figure 3A), as well as the dorsal–lateral compartment of mutant somite (Figure 3B). Consequently, this combination of zone expansion and elevated levels results in a ~3.5-fold increase in Pax7 protein in E9.5 *Pax3^neo/∆5^* embryos relative to control (Figure 3E). Further, RT-PCR analysis verified an elevated level of *Pax7* mRNA in E9.5 *Pax3^neo/∆5^* (Figure 3F). As *Pax* genes are believed to have evolved via duplication from four corresponding ancestral genes [8], we examined whether Pax7 upregulation is a specific response to Pax3 reduction. Using RT-PCR, we measured the expression levels of other *Pax* sub-group members (Figure 3G). Consistent with their evolutionarily divergent relationships, all *Pax* members from other groups (1, 2, 5, 6, 8 and 9) are expressed at similar levels between *Pax3^neo/∆5^* and the wildtype, except *Pax7* which is elevated in *Pax3^neo/∆5^* mutants (Figure 3G). These data confirm compensatory transcriptional and translational upregulation of closely-related Pax7 in *Pax3^neo/∆5^* mutants, and the specific activation of ectopic Pax7 expression in the hypomorphs provides further evidence to support the proposed functional linking between Pax3 and Pax7 during evolution [8].

### 3.3. Pax7 Plays a Functional Genetic Compensation Role in Heart and Neural Tube Development

**Figure 4:**
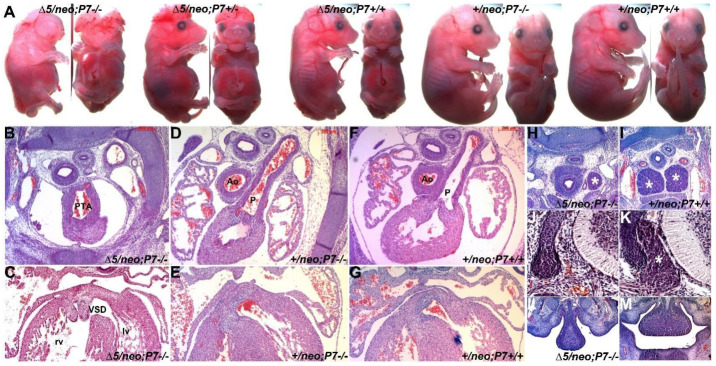
Concomitant reduction in Pax7 expression triggers exacerbated neural tube and heart defects in *Pax3^∆5/neo^* mutants. (**A**) Whole E17.5 fetal lateral (left) and frontal (right) views of indicated genotypes. Loss of one *Pax7* allele exacerbates the cranial exencephaly and caudal spina bifida neural tube closure defects in *Pax3^∆5/neo^*/*Pax7^Δ5/+^* mutants and loss of both *Pax7* alleles results in additional mid-face clefting and complete failure of cranial neural tube closure in *Pax3^∆5/neo^*/*Pax7^Δ5/Δ5^* mutants. (**B**–**G**) Histology reveals E15.5 *Pax3^∆5/neo^*/*Pax7^Δ5/Δ5^* mutants exhibit fully penetrant PTA (**B**) and VSD defects (**C**) when compared to *Pax3^+/neo^*/*Pax7^Δ5/Δ5^* and *Pax3^+/neo^*/*Pax7^+/+^* mutants. Note that *Pax3^Δ5/neo^*/*Pax7^Δ5/Δ5^* hearts only have a single abnormal outflow tract exiting the right ventricle (**B**) and that there is a membranous interventricular sepal defect resulting in aberrant communication between the left (lv) and right (rv) ventricles (**C**), while unaffected hearts have a separate aorta (Ao) exiting the left ventricle and a pulmonary trunk (p) exiting the right ventricle (**D**,**F**), and there is no communication between the left and right ventricles (**E**,**G**). (**H**–**K**) The E15.5 neural crest-derived *Pax3^∆5/neo^*/*Pax7^Δ5/Δ5^* thymus (* in **H**) and dorsal root ganglion (**J**) are malformed, as the thymus is smaller and often missing a lobe and the DRGs are absent, compared to control littermate bi-lobed thymus (* in **I**) and DRGs (* in **K**). (**L**,**M**) The *Pax3^∆5/neo^*/*Pax7^Δ5/Δ5^* tongue is hypoplastic and lacks most of the musculature and there is a severe loss of upper jaw (**L**) when compared to wildtype littermates. Scale (**B**–**G**,**H**,**I**,**L**,**M**) 200 μm; (**J**,**K**), 100 μm.

The observations that *Pax3^neo/Δ5^* mutants do not exhibit any of the typical *Pax3*-deficent cardiovascular defects and that Pax7 ectopic expression is a specific response to 90% Pax3 reduction in *Pax3^neo/∆5^* prompted us to test the biological significance of this augmented Pax7 expression. To achieve this goal, we generated *Pax3^neo/∆5^* embryos bearing either one (+/−) or both (−/−) *Pax7^Δ2^* null alleles [27], enabling us to test the potential impacts of 50% or 100% reduction in Pax7 dosages within the *Pax3^neo/∆5^* background. Using Western analysis of E11 whole-embryo lysates, we confirmed a gene dosage-responsive reduction in Pax7 protein levels. Relative to 100% in the wildtype littermate control, *Pax7^Δ2/+^* and *Pax7^Δ2/Δ2^* embryos (*n* = 3 of each) express 50% and 0% Pax7 protein, respectively (data not shown). Although the *Pax7^Δ2^* null allele is different from the reported mutant *Pax7^Δ1^* allele [11], we observed that *Pax7^Δ2^*^/*Δ2*^ mutants alone do not impact normal limb, neural tube or heart development (Figure 4A), which is consistent with previous reports [11]. Surprisingly, *Pax3^neo/∆5^/Pax7^Δ2/+^* mutants were 100% viable *in utero* (*n* = 16 litters). However, the removal of one *Pax7* allele was sufficient to significantly increase the frequency of neural tube defects (from 66% *Pax3^neo/∆5^/Pax7^+/+^* having spina bifida and/or exencephaly to 95% (*n* = 47/49) of *Pax3^neo/∆5^/Pax7^Δ2/+^* having spina bifida and/or exencephaly). Moreover, the loss of both *Pax7* alleles resulted in fully penetrant neural tube defects (100% (*n* = 24/24) *Pax3^neo/∆5^/Pax7^Δ2/Δ2^* having spina bifida and exencephaly). Additionally, the loss of both *Pax7* alleles caused an exacerbated fully penetrant cranial neural tube defect (Figure 4A) as well as loss of upper jaw structures (100% (*n* = 24/24) in *Pax3^neo/∆5^/Pax7^Δ2/Δ2^* mutants; Figure 4L) that was not seen in either *Pax7* null, *Pax3* null or hypomorph mutants (both *Pax3^neo/neo^* and *Pax3^neo/∆5^*). Further, in contrast to *Pax3^neo/∆5^/Pax7^Δ2/+^* mutants, the majority of *Pax3^neo/∆5^/Pax7^Δ2/Δ2^* mutants also exhibit edema and appear to die between E13.5 to E16.5 (*n* = 16 litters). Thus, despite Pax7 being shown to be robustly expressed in facial structures, these compound mutants now reveal a potential unanticipated functional compensatory functional role for Pax7 during craniofacial morphogenesis and frontonasal neural tube closure.

Although Pax7 is itself not thought to play a role within cardiac neural crest nor outflow tract development, the presence of edema is suggestive of *in utero* heart failure [33]. Thus, we histologically examined E15.5 serial sections and found that *Pax3^neo/∆5^/Pax7^Δ2/+^* mutants all exhibit outflow tract defects. Specifically, *Pax3^neo/∆5^/Pax7^Δ2/+^* mutants exhibit 57% (*n* = 4/7) PTA and 45% (*n* = 3/7) DORV, and 100% (*n* = 7/7) also contain concomitant VSDs, whilst *Pax3^neo/∆5^/Pax7^Δ2/Δ2^* mutants have a 100% (*n* = 7/7) incidence of PTA and concomitant VSDs (Figure 4B,C). These data are in contrast to *Pax3^neo/∆5^/Pax7^+/+^, Pax3^neo/+^/Pax7^Δ2/+^* and *Pax3^neo/+^/Pax7^Δ2/Δ2^* mutants that all have normal outflow tract with separate aorta and pulmonary trunks and a septated interventricular septum (Figure 4D–G). Taking advantage of the *neo* allele, we also investigated the impact of the loss of Pax7 on heart development within the *Pax3^neo/neo^* background. While deficiency of Pax7 in *Pax3^neo/neo^* mutants results in full penetrance of exencephaly, heart development was unaffected (*n* = 5/5) in *Pax3^neo/neo^/Pax7^Δ2/Δ2^* (data not shown). Thus, this differential impact of *Pax7* loss suggests the threshold Pax3 expression level required is set at around 20%, where a Pax3 reduction lower than 20% is sufficient to a trigger a heart defect. Given the critical role the cardiac neural crest plays within outflow tract development and the finding that ectopic Pax7 is expressed in the neural crest domain within mutant neural tubes (see Figure 3), we examined other neural crest derivative tissues. As expected, *Pax3^neo/∆5^/Pax7^Δ2/Δ2^* mutant thymus is reduced in size to such an extent that either it was detected only on a limited number of sections or only had a single lobe (Figure 4H). Likewise, *Pax3^neo/∆5^/Pax7^Δ2/Δ2^* mutant DRGs are absent (Figure 4J). Thus, these compound *Pax3* hypomorph data show that multi-lineage neural crest, neural tube and cardiac phenotypes are triggered by a greater than 20% Pax3 reduction and have uncovered a hitherto unknown dosage-dependent neural crest, neural tube and cardiogenic abilities for Pax7.

### 3.4. Pax7-Expressing Cell Lineage Is Ectopically Present in Neural Crest Derivative

**Figure 5:**
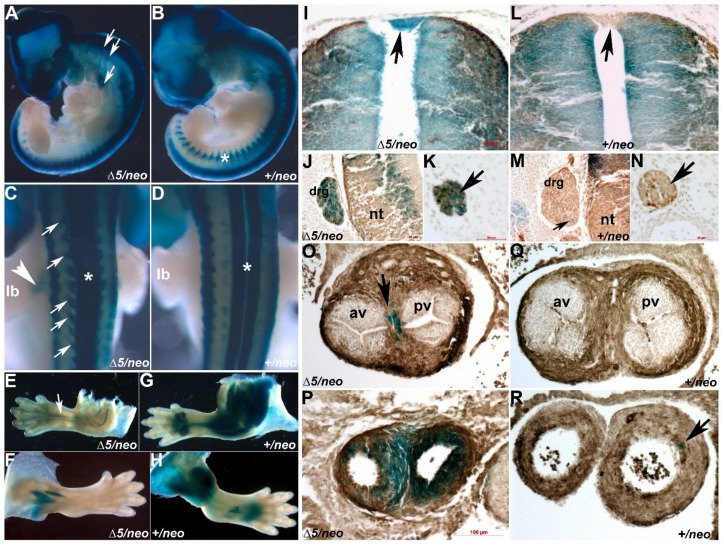
Pax7^Cre^/R26r lineage mapping reveals ectopic Pax7-marked cells colonize Pax3 locations. (**A**–**D**) Left lateral (**A**,**B**) and dorsal (**C**,**D**) views of *Pax3^∆5/neo^*/*Pax7^Cre^/R26r^LacZ^* (**A**,**C**) and control (**B**,**D**) X-Gal stained E11 embryos, illustrating enhanced and ectopic *Pax7*-marked neural crest emigration towards the pharyngeal arches (**A**, double arrows) and within the 4/6th circumpharyngeal crest (**A**, single arrow), when compared to control *Pax3^+/neo^* littermate (**B**). Additionally, note reduced *Pax7^Cre^* labeling of myocytes in mutant somites compared to control (* in **B**). Dorsal view revealed *Pax7^Cre^-*marked cells ectopically colonize the *Pax3^∆5/neo^* DRGs (**C**, small arrows), the neural tube roofplate (* in **C**) and PNS neural crest emigrating into mutant fore-limbs (**C**, large arrow, lb) but not control limbs or roofplate (* in **D**). (**E**–**H**) X-Gal staining of E15.5 *Pax7^Cre^*-marked cells in in *Pax3^∆5/neo^* fore- and hind-limbs (**E**,**F**) compared to control limbs (**G**,**H**) which exhibit robust staining of muscle lineages. Note ectopic *Pax7^Cre^* positive labeling of PNS is obvious in only the *Pax3^∆5/neo^* mutant fore-limb (**E**, arrow) due to severe hypoplastic musculature, whereas LacZ staining is absent in *Pax3^∆5/neo^* hind-limbs (**F**). (**I**–**N**) Histology and neuron-specific β3 Tubulin IHC (brown DAB-positive cells) confirmed E11 ectopic lacZ labeling of *Pax3^∆5/neo^* dorsal most region of the neural tube roofplate (**I**,**L**, arrow), the DRGs (**J**) and strong bilateral staining was evident in the developing sympathetic ganglia (**K**, arrow), when compared to control roofplate and DRG which do not contain lacZ cells (arrows in **M**,**N**). Note while mutant DRGs are ~100% LacZ labeled, the control DRG only has a single lacZ cell in this section (**M**, small arrow). (**O**–**R**) Transverse sections through αSmooth muscle actin IHC (brown DAB-positive cells) in outflow tract regions in *Pax3^∆5/neo^* mutant (**O**,**P**) and *Pax3^+/neo^* control (**Q**,**R**) hearts. Significant ectopic LacZ cells are present in both the *Pax3^∆5/neo^* valve (small arrow in **O**) and more anterior outflow tract vessel (**P**) planes. Note robust *Pax7^Cre^*-marked populations colonize *Pax3^∆5/neo^* outflow tract septum between the aortic (av) and pulmonary (pv) valves, as well as the smooth muscle of the great arteries, but a few isolated LacZ cells were found in more anterior planes in controls (**R**, arrow). Scale (**I**–**N**), 50 μm; (**O**–**R**), 100 μm.

Given that our studies unmasked a compensatory cardiogenic role of Pax7 in *Pax3^neo/∆5^/Pax7^Δ2/Δ2^* mutants, we theorized that ectopic Pax7 compensates for reduced Pax3 levels in the dorsal-most neural tube, and that this ectopic Pax7 sustains sufficient neural crest morphogenesis to facilitate normal outflow tract septation. To test this hypothesis, we used *Pax7^Cre^/R26r^LacZ^* lineage mapping to examine spatiotemporally how Pax7 was aberrantly expressed in *Pax3^neo/∆5^* hypomorphs. As Pax3 and Pax7 can both exhibit dosage-dependent defects, we initially had to determine whether the *Pax7^Cr^*^e^ allele both recapitulates wildtype *Pax7* expression and then subsequently ensured both Pax7 and Pax3 levels were unaffected. To validate that neither of these potential caveats were of concern, we used a *Cre* knocked in at 3′UTR of *Pax7* gene locus that expresses both Pax7 and Cre under endogenous Pax7 promotor [29]. As expected, *Pax7^Cre^/R26r^LacZ^* lineage mapping faithfully recapitulates endogenous *Pax7* expression within control E9.5 embryonic hindbrain neural tube, forehead and pharyngeal arches, and *Pax7*-derived cells are expressed in E13 craniofacial, muscle and defined neural tube cells (Supplemental Figure S2A–C). Intriguingly, *Pax7^Cre^* expression is absent from rhombomeres 3 and 5 that do not give rise to emigrating neural crest [34] and is only present in neural -rest-producing rhombomeres 2 and 4. Further, *Pax7^Cre^/R26r^LacZ^* lineage mapping detected a small population of *Pax7-*derived cells present in the wildtype E13 outflow tract (Supplemental Figure S2D). Supportively, *Pax7^Cre/Cre^, Pax7^Cre/+^* and wildtype E10 embryos all express equivalent levels of Pax7 and Pax3 proteins (Supplemental Figure S2E). Given that Pax3 and Pax7 are remain unaffected, this *Pax7^Cre^* knock-in allele is an appropriate model to enable mapping of *Pax7*-derived lineages associated with a hypomorphic reduction in Pax3. As anticipated given the known somite deficiencies in *Pax3* nulls [2,14,16], *Pax7^Cre^-*marked muscles are largely absent in E11 *Pax3^neo/∆5^* hypaxial dermomyotome and limbs compared to control littermates (Figure 5A–H). However, in agreement with IHC showing ectopic Pax7 protein within the dorsal-most *Pax3^neo/∆5^* neural tube roofplate (see Figure 5C,I), there is similar *Pax7^Cre^* indicator expression in the dorsal-most neural tube (Figure 5I,J). Similarly, ectopic *Pax7*-derived LacZ cells were observed in both the E11 migratory cardiac neural crest (Figure 5A), neural-crest-derived DRGs (Figure 5A,C,J) and in peripheral nervous system (PNS) neural crest-derived sympathetic ganglia (Figure 5C,K). Further, in E15.5 *Pax3^neo/∆5^* hypomorphs, we found robust ectopic *Pax7^Cre^-*derived cells present in mesenchymal cells of the outflow tract and smooth muscle cells of the great arteries (Figure 5O,P) but are absent from wildtypes (Figure 5O,R). Additionally, Schwann cells in the peripheral nervous system (PNS) were ectopically *Pax7^Cre^*-labeled in *Pax3^neo/∆5^* forelimbs but not hindlimbs nor wildtype fore- or hind-limbs (Figure 5E,F). Similarly, enteric neural crest cells of the digestive tract were also ectopically labeled in E15.5 *Pax3^neo/∆5^* mutants (data not shown). Neuronal and smooth muscle cell identities of *Pax7* derivatives were confirmed via neuron-specific β3-tubulin or α—smooth muscle actin IHC. β3-tubulin marker overlaps ectopic and endogenous *Pax7^Cre^* LacZ expression in *Pax3^neo/∆5^* but not wildtype littermates (Figure 5J–N), and α—smooth muscle actin overlaps ectopic *Pax7^Cre^* LacZ expression in *Pax3^neo/∆5^* outflow tract but not wildtype littermates (Figure 5O–R). Thus, consistent with the onset of endogenous Pax7 being cranial to caudal (see Supplemental Figure S2), ectopic *Pax7* is mainly activated in more anterior neural crest *Pax3^neo/∆5^* lineages. Combined with the embryonic data, the continued presence of ectopic *Pax7*-derived descendent neural crest indicates that the ectopic Pax7 lineage is persistent rather than transient and that ectopic *Pax7*-marked cells can colonize endogenous Pax3 neural crest/dorsal-most neural tube expression domains only when Pax3 levels are very low.

### 3.5. Pax3/7 Double Nulls Exhibit Exacerbated Defects

**Figure 6:**
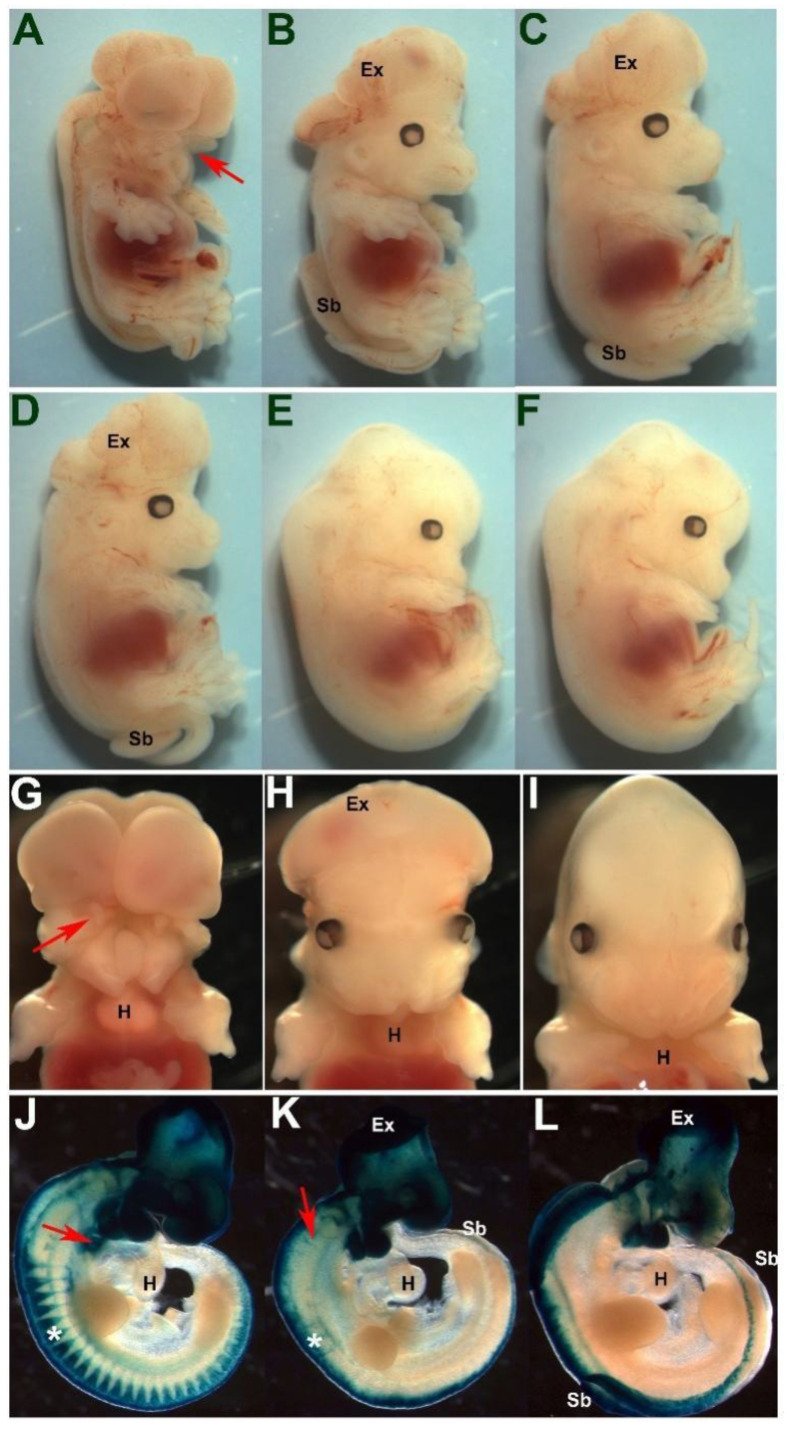
*Pax3/7* double nulls exhibit exacerbated defects. (**A**–**F**) Whole E14.5 fetal lateral (left) views of different combinations of *Pax3* and *Pax7* allelic status. *Pax3^∆5/Δ5^*/*Pax7^Δ5/Δ5^* double nulls exhibit craniorachischisis, contiguous with craniofacial dysplasia (**A**, red arrow), but *Pax3^∆5/Δ5^*/*Pax7^Δ5/+^* (**B**), *Pax3^∆5/Δ5^*/*Pax7^+/+^* (**C**) and *Pax3^∆5/+^*/*Pax7^Δ5/+^* (**D**) mutants only exhibit exencephaly (Ex) and spina bifida (Sb). (**G**–**I**) Frontal views demonstrate that *Pax3^∆5/Δ5^*/*Pax7^Δ5/Δ5^* double nulls exhibit frontal clefting (**G**, arrow) but neither *Pax3^∆5/Δ5^*/*Pax7^+/+^* (**H**) and *Pax3^+/+^*/*Pax7^Δ5/Δ5^* (**I**) null facial morphogenesis remained unperturbed. (**J**–**L**) *Wnt1^Cre^/R26r^LacZ^* lineage mapping of E11 neural crest cells revealed robust staining (blue) in *Pax3^+/+^*/*Pax7^Δ5/Δ5^* neural crest-derived DRGs (**J**, *), emigrating neural crest and cardiac neural crest in the circumpharyngeal crest adjacent to the outflow tract (**J**,**K**, red arrows), as well as the craniofacial and neural tube cells. Note this pattern is identical; to that in wildtype embryos [15]. However, lacZ staining of *Pax3^∆5/Δ5^*/*Pax7^Δ5/+^* DRGs and emigrating cardiac neural crest is severely diminished (**K**), and lacZ staining of *Pax3^∆5/Δ5^/Pax7^+/+^* DRGs and emigrating cardiac neural crest is totally absent (**K**). Further, spina bifida neural tube closure defect is more extensive in *Pax3^∆5/Δ5^*/*Pax7^Δ5/Δ5^* double nulls.

To further assess genetic redundancy and/or functional genetic compensation and determine whether complete ablation of Pax3 and Pax7 is more severe than the defects observed in *Pax3^neo/∆5^/Pax7^Δ2/Δ2^* mutants, we generated *Pax3/7* double mutants on a defined genetic background (Figure 6). Significantly, *Pax3/7* double knockouts live up to ~E14 but are all dead by E15.5 (*n* = 6/6). Further, they exhibit a complete failure of neuropore closure along the entire neural tube (Figure 6A), including lack of closure at neural tube closure sites 1–3 [35]. This results in the most severe neural tube defect, craniorachischisis, in which the brain and spinal cord remain open. Allied to this, double *Pax3/7* nulls exhibit contiguous craniofacial dysplasia phenotype with fully penetrant frontal clefting (Figure 6A,G), resulting from failed anterior (cranial) and posterior (caudal) closure and encompasses both failure within both the primary and secondary neurulation phases [35]. Moreover, as *Pax3/7* double null mutants do not die earlier than *Pax3* nulls, the observed exacerbated *Pax3/7* double null craniofacial defects suggest overlapping functional compensation. Thus, compete loss of all four paralogous *Pax3* and *Pax7* alleles results in more severe neural tube defects, suggesting that the 10% Pax3 remaining in *Pax3^neo/∆5^/Pax7^Δ2/Δ2^* mutants was sufficient for anterior closure of neuropore 3 but not more caudal neuropore closure.

As *Pax3^neo/∆5^/Pax7^Δ2/Δ2^* mutants already exhibit fully penetrant PTA with concomitant VSDs, we examined the E14 Pax3/7 double knockout cardiovascular system. As expected, 100% double *Pax3/7* nulls exhibit PTA with concomitant VSDs congenital heart defects (*n* = 6/6) and are edematous. Moreover, we also examined whether neural crest pathogenesis, and more specifically, cardiac neural crest emigration to the outflow tract were more comprised in double *Pax3/7* nulls. Using *Wnt1^Cre^/R26r^LacZ^* lineage mapping of pre-migratory and migratory neural crest cell lineages [28], we confirmed that *Pax3^∆5/∆5^*/Pax7^∆^^2/+^ neural crest emigration is already severely deficient (*n* = 3/3) compared to the wildtype (*n* = 5/5), double heterozygous and *Pax3^+/+^*/Pax7^∆^^2/+^ littermates (Figure 6J,K) that all have normal hearts. However, *Pax3/7* double nulls (*n* = 4/4) exhibit an exacerbated lack of Pax3/7-derived neural crest emigration to the embryonic heart (Figure 6L). Further, while *Pax3^∆5/∆5^/Pax7^Δ2/+^* null DRGs were mainly diminished, some were still recognizable (Figure 6K), *Pax3/7* double null DRG were completely deficient (Figure 6L). Thus, the loss of all four *Pax3* and *Pax7* alleles results in more severe neural crest defects, suggesting that the 10% Pax3 remaining in *Pax3^neo/∆5^/Pax7^Δ2/Δ2^* mutants was sufficient for limited neural crest emigration but insufficient for cardiac neural crest colonization to mediate outflow tract septation.

### 3.6. Ectopic Pax7-Expressing Lineage Is Essential for Heart and Craniofacial Morphogenesis

**Figure 7:**
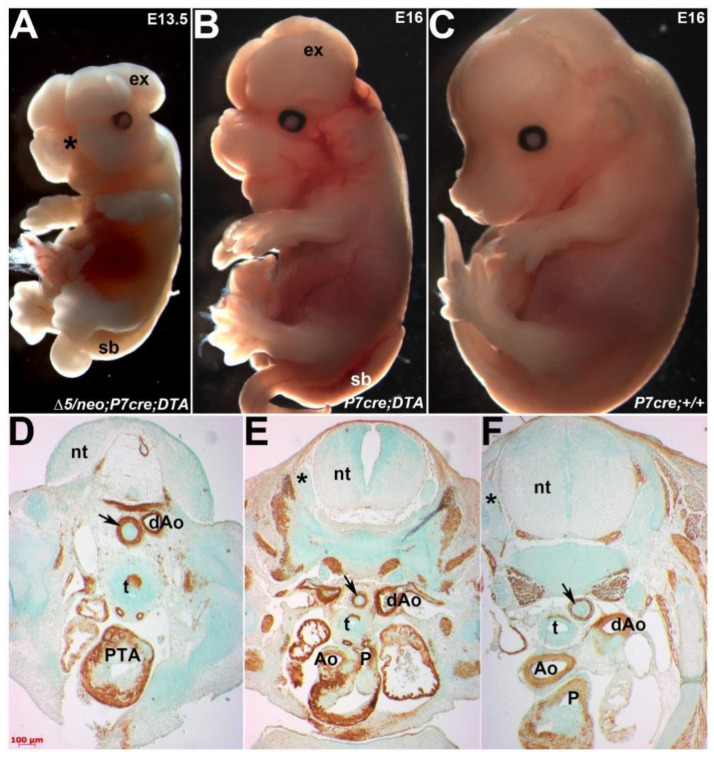
Genetic cell ablation of *Pax7* lineage in only *Pax3^∆5/neo^* mutants results in congenital heart defects. (**A**–**C**) Gross phenotypes following Cre/loxP-mediated genetic cell ablation in E13.5 *Pax3^∆5/neo^*/*Pax7^Cre^/R26^-EGFP-DTA^* (**A**), E16 *Pax7^Cre^/R26^-EGFP-DTA^* (**B**) compared to E16 *Pax3^∆5/neo^* non-DTA control (**C**). As all *Pax3^∆5/neo^*/*Pax7^Cre^/R26^-EGFP-DTA^* ablation mutants die by E15.5, we examined only viable E13.5 embryos. Note *Pax7^Cre^*-mediated ablation in both *Pax3^∆5/neo^* mutant and control backgrounds causes exencephaly (ex), spina bifida (sb), but that mid-face clefting (* in **A**) is only present in *Pax3^∆5/neo^* ablation mutants. As expected, presence of *Pax7^Cre^* alone (**C**) does not cause any abnormalities. (**D**–**F**) IHC using αSmooth muscle actin revealed enhanced muscular hypoplasia in *Pax3^∆5/neo^* mutants (**D**) and that only *Pax7^Cre^* mediated cell ablation results lack of outflow tract septation in *Pax3^∆5/neo^* ablation mutants (**D**) but not Pax7Cre ablation mutants (**E**). Note *Pax7* lineage-ablated *Pax3^+/neo^* hearts only have a single abnormal PTA exiting the right ventricle (**D**) but *Pax7* lineage-ablated controls have unaffected hearts with separate aorta (Ao) and pulmonary trunks (p) exiting the heart. Additionally, note that the DRGs (* in **E**) are intact in *Pax7* lineage-ablated controls and wildtype (* in **F**) but are absent in *Pax7* lineage-ablated *Pax3^+/neo^* mutants (**D**). Small arrows in (**D**–**F**) indicate similar planes through the esophagus. Abbreviations: nt, neural tube; dAo, descending aorta. Scale (**D**–**F**), 100 μm.

The persistence of *Pax7^Cre^*-marked cells in both the dorsal-most neural tube, multiple neural-crest derived structures and consequently the hypomorphic outflow septum prompted us to examine their biological requirement during development. To this end, we employed Cre-mediated genetic cell ablation via diphtheria toxin fragment-A (DTA) expression [30]. Cre-mediated activation of DTA kills cells via apoptosis by interfering with the RNA translation machinery in each individual cell. This system is highly autonomous as only cells that express the DTA are ablated without any effects on neighboring non-Cre expressing neural crests [31]. We crossed *Pax3^neo/∆5^* mice carrying the *Pax7^Cre^* knock-in allele with *R26r^DTA^* mice, resulting in compound controls, *Pax7^Cre^/R26r^DTA^* mutants on a *Pax3* wildtype background and hypomorphic *Pax3^neo/∆5^*/*Pax7^Cre^/R26r^DTA^* mutants. Significantly, *Pax7^Cre^/R26r^DTA^* ablation mutants (*n* = 8/8) exhibit exencephaly and spina bifida along with craniofacial defects, whereas *Pax3^neo/∆5^/Pax7^Cre^/R26r^DTA^* ablation mutants (*n* = 4/4) exhibit exencephaly and spina bifida but also complete facial clefting (Figure 7A,B). Moreover, all the *Pax3^neo/∆5^/Pax7^Cre^/R26r^DTA^* ablation mutants exhibit PTA with VSDs and died by E15.5, even though *Pax7^Cre^/R26r^DTA^* ablation mutants have normally septated hearts (Figure 7D,E) and live until birth. In agreement with control lineage mapping (see Supplemental Figure S2) showing robust craniofacial and neural tube expression but only a small clump of cells in outflow tract, *Pax7^Cre^-*mediated cell ablation on a *Pax3* wildtype background did not result in any detectable heart defects, while ablation of *Pax7*-expressing cells did affect cranial structures. Similarly, neural-crest-derived DRGs were only hypoplastic in ablation mutants on the hypomorphic background (Figure 7D). Thus, these ablation data indicate that the persistent presence of ectopic *Pax7^Cre^-*marked cells is vital for intact neural crest and outflow morphogenesis and that these *Pax7-*expressing cells can directly compensate for diminished Pax3 levels. Further, these results suggest that ectopic Pax7 can partially fulfill Pax3’s requirements during neural crest delamination and neural fold closure when Pax3 levels are very low (10%), but Pax7 cannot compensate for Pax3 function when Pax3 is absent (0%). Thus, we have identified compensatory upregulation of closely-related Pax7 in *Pax3* hypomorphic mutants, demonstrating genetic functional genetic compensation and a hitherto unappreciated ability of Pax7 within neural tube, neural crest and cardiovascular biological processes.

## 4. Discussion

To elucidate why *Pax3^neo/neo^* (20% Pax3) and *Pax3^neo/Δ5^* (10% Pax3) hypomorphs only partially phenocopied systemic *Pax3* nulls and why only select tissues derived from *Pax3*-expressing progenitors are affected but others are impervious to 10% Pax3 levels, we employed several combinatorial transgenic models and carried out molecular analysis of Pax3 and Pax7 spaciotemporal expression. Using compound mutants, lineage mapping and genetic cell ablation approaches enabled us to determine that paralogous Pax7 plays a direct functional genetic compensational role during neural tube closure, several neural crest lineage’s development and outflow tract morphogenesis within *in utero* hearts. Herein, we demonstrated that endogenous Pax7 is not only upregulated but is also ectopically induced within Pax3-expression domains in *Pax3^neo/Δ5^* hypomorphs. Thus, we show that ectopic Pax7 is absolutely required and that Pax7 can play an overlapping compensatory functional role during refractory 10%-expressing *Pax3^neo/Δ5^* cardiac neural crest morphogenesis and craniofacial development to ensure hypermorphic *in utero* survival.

Outflow tract defects are a significant cause of morbidity and mortality in newborn infants and are amongst the most highly encountered congenital defects [12,13,15]. Intriguingly, in non-mammalian species *Xenopus* Pax3/7 neural crests, *Pax3* appears as the earliest neural border-specific marker [36], and in chick Pax3/7 neural crests, a similar pattern is assumed by *Pax7* [37]. Correspondingly, despite mammalian *Pax7* homozygous mutants living up to weaning without any sign of neural tube or heart defects [11], Pax7 being excluded from the dorsal-most neural crest generating regions of the early neural tube and *Pax7* being expressed later in development than *Pax3* [11], *Pax7^Cr^*^e^-marked decedents were shown to migrate along known neural crest cells pathways and to colonize the mouse heart [18]. Suggestively, we previously demonstrated that *Pax3^neo/neo^* hypomorphs express ectopic Pax7 at sites where endogenous Pax3, but not Pax7, is normally expressed [2]. Thus, although several lines of evidence have demonstrated that *Pax7* is present in cardiac neural crest and fetal hearts, it was unclear what potential the Pax7-expressing lineage has and what functional role it may play. Consistent with lineage mapping data, we confirmed that wildtype outflow is colonized by a persistent small group of *Pax7^Cre^*-marked cells and that *Pax7^Cre^*-meditated genetic ablation verified that Pax7-expressing derivative cardiac neural crest cell lineage is required for normal outflow separation within *Pax3^neo/Δ5^* hypomorphic hearts. Moreover, other hypomorphic neural crest lineages are similarly ‘rescued’ as DRGs, Schwann and sympathetic ganglia each contain significant colonization via ectopic *Pax7^Cre^*-marked cells. Our data suggest that this happens because Pax3 can negatively regulate Pax7 [2,16,22,23], and the *Pax3^neo/Δ5^* reduction in Pax3 levels likely results in sufficient ectopic and induced Pax7 upregulation (~3.5x fold) within Pax3-expressing lineages. However, when Pax3 is totally absent and thus unable to regulate Pax7, there is no Pax7-mediated compensatory response within the dorsal-most neural tube and thus *Pax3* nulls fail to generate sufficient cardiac neural crest required for outflow tract separation and *in utero* survival. This suggests a mechanism whereby *Pax3^neo/Δ5^* cardiac neural crest lineage is plastic and can respond to residual Pax3 along with ectopic Pax7 to undergo adequate neural crest emigration and colonization of the heart. As Pax3 is known to play a role in cell survival [16] and genetic ablation of p53-dependent apoptosis prevents neural tube defects in *Pax3* nulls [38], residual Pax3 may be required for the survival of the neural crest cells that ectopically express Pax7. Additionally, our findings that ectopic Pax7-marked cells can colonize endogenous *Pax3* neural crest/dorsal-most neural tube expression domains could also suggest cell compensation, in addition to the gene compensation between the two Pax paraloges. However, future more regionally restricted *Cre* lineage mapping approaches will be required to verify if cell compensation also occurs in *Pax3* hypomorphs. Alternatively, structurally related Pax3 and Pax7 transcription factors may both be able to similarly regulate the same set of downstream neural crest effectors, and thus the combination of residual Pax3 and induced Pax7 is adequate to sustain neural crest morphogenesis. Although *bona fide* Pax3 and Pax7 neural crest targets remain unknown [39,40], both Pax3 and Pax7 exhibit conserved *cis*-acting transcription repression domains [41] and both may bind identical DNA motifs [42]. For instance, the *Dbx1* neural tube homeobox can sense the levels of *Pax3* and *Pax7* transcriptional activity [43] and only *Pax3/7* double null embryo neural folds/tubes exhibit expanded *Engrailed* and absent *Wnt4* expression [20]. Moreover, Pax3 and Pax7 are also both associated directly with β-catenin and Wnt effector complex [44,45] and *Msx2* and *Msx1/2* double nulls both exhibit dorsal expansion of *Pax7* in the dorsal-most neural tube [46,47]. Indeed, the deficiency of cardiac neural crest development in *Pax3*-null mice is thought to be caused by upregulation of *Msx2*, as *Msx2/Pax3* double nulls prevented outflow tract defects routinely observed in single *Pax3* nulls [46]. However, it remains to be determined whether Pax3 and Pax7 directly interact to either repress or induce common neural crest targets or indirectly via Pax3 regulating a mediator that subsequently negatively modulates Pax7 expression and/or is required for neural crest cell survival. Given that our prior lineage-restricted *Pax3* conditional knockout studies revealed that Pax3 function is not required in cardiac neural crest cells once they have initiated emigration from the neural tube and is the only cell autonomously required briefly in the early neural folds within pre-somitic ~E8 embryos [13], this indicates that the observed ectopic Pax7 induction in the *Pax3^neo/Δ5^* roofplate is an early event and is sufficient to facilitate neural crest emigration. Thus, descendants from Pax7-expressing cells are not only structurally present in normal hearts and multiple other neural crest-derived tissues, but they can also functionally compensate in *Pax3* hypomorphs expressing 10% Pax3 levels in vivo.

Another interesting finding is the unanticipated severe craniofacial morphogenesis defects observed in compound Pax3/7 mutants. Indeed, craniofacial defects such as cleft lip and cleft palate are among the most common of all birth defects. Our data revealed that despite *Pax3* null and *Pax7* null facial derivatives of neural crest are largely spared [12,18,20], *Pax3^neo/Δ5^* hypomorphic mice expressing 10% Pax3 levels exhibit severe facial morphogenesis defects when compensatory Pax7 is removed. Moreover, *Pax3/7* double null mutants similarly exhibit exacerbated craniofacial defects compared to *Pax3* nulls, supporting our hypothesis of overlapping functional compensation. Although our analysis was not able to differentiate whether the *Pax3^neo/Δ5^* cranial neural crest or neural tube closure were the primary causes of the exacerbated craniofacial defects, these data demonstrated that Pax7 can functionally compensate for diminished Pax3 levels. This indicates that, like compound *Pax3/7*, heterozygotes that display partially penetrant congenital hydrocephalus [19], *Pax3^neo/Δ5^* that lack compensatory Pax7 revealed that cranial dysmorphogenesis can be caused via collective Pax3 and Pax7 deficiencies. Indeed, murine *Pax3/7* gene deficiencies and dosage alterations are known to play essential safeguard functions against environmental stress-induced birth defects [48], persistent ectopic transgenic expression of Pax3 in cranial neural crest cells can result in a cleft palate [49], and orofacial cleft birth defects are associated with PAX7 mutations in patients [50]. Although the mechanism whereby Pax3 and Pax7 directly interact in hypomorphs within craniofacial development remain unknown, these data demonstrate a direct relationship between these two paralogs. Moreover, as Pax3 is specifically expressed within the neural tube both before and after neural tube closure, whilst Pax3 is only transiently expressed in cardiac neural crest progenitors and is absent from within the heart, these distinct spatiotemporal expression patterns most likely account for the divergent sensitivity to Pax3 suppression and give rise to the disparate cardiovascular and neural tube closure defects observed. Thus, this novel *Pax3^neo/Δ5^* mutant mouse provides a genetically defined model to begin to understand how genetic, and possibly environmental, factors could affect Pax3 and Pax7 interactions, and how they may impact the prevalence of congenital craniofacial defects.

Within our mouse model systems, we demonstrated that both compensatory Pax gene dosage and Pax gene functional genetic compensation occur. Presently, the molecular foundation underlying Pax3/7 dosage requirements are unknown, but based on haploinsufficiency, it is generally assumed that Pax proteins act within a concentration range sensitive to 2x-fold changes [51]. Our findings that Pax3 is reduced to 10% in *Pax3^neo/Δ5^* and that Pax7 is ectopically induced and upregulated 3.5x-fold support this assumption and further advocate that Pax7 is autoregulated in most *Pax3*-expressing cells in response to substantial Pax3 protein losses. Further, *Pax3/7* patterning of intermediate spinal interneurons has been shown to be due to inhibition of multiple alternative fates rather than relying on binary decisions in the neural tube [43], signifying that dorsal neural tube cell fates require sufficient Pax3/7 translational repression for normal morphogenesis to occur. This aspect is supported but the demonstration that knock-in of the *Pax7* coding region can substitute for the loss of Pax3 function, as replacement of diminished Pax3/7 translational repressor function in *Pax3* nulls was sufficient to enable normal dorsal neural tube, neural crest and somitic development in *Pax3^Pax7-ILZ/Pax7-ILZ^* mutants [21]. Significantly, *Pax3* and *Pax7* are the only *Pax* family members to encode the paired-box domain, paired-type homeo-domain and octapeptide sequences, and only these paralogs can functionally compensate for each other. Tellingly, despite Pax8 expression in the neural tube in a domain ventral to that of Pax3, *Pax8* (which lacks a full paired-type homeo-domain) cannot replace Pax3 function in the dorsal neural tube [52]. Indeed, the *Pax3/7* homeo-domains both physically interact and can cooperate with paired-domain DNA-binding ability [53]. Thus, as both *Pax3^neo/Δ5^* hypomorphs and *Pax3^Δ5/Δ5^* nulls were generated via insertion of either a neomycin cassette [2] or creation of a premature stop codon in the homeodomain [26], this indicates that reduced and/or loss of Pax3 homeodomain function is likely primarily responsible for the observed *Pax3^neo/Δ5^* defects. As genetic redundancy is generally thought to arise from genetic duplication or from functional genetic compensation via upregulation of compensating genes, the presence of Pax3/7 paralogs indicate that increased dosage of Pax3/7 gene products may be beneficial in response to different genetic insufficiencies or environmental stress conditions. Furthermore, the finding that Pax7 is usually excluded from Pax3-expressing neural crest specification and emigration domains [7.9,10] suggests that it is the ectopic Pax7 expression that is key to meditating resistance to diminished Pax3 levels. As ectopic expression is usually thought to represent abnormal gene expression in a cell type or developmental stage in which it is not known to have a function, as opposed to overexpression, which implies increased expression beyond the norm [54], our data support an abnormal ectopic expression mechanism in *Pax3* hypomorphs. Although the role of transcriptional suppressors and/or enhancers in regulating overexpression is well documented, the effectors responsible for ectopic expression are less clear [55]. However, prior data [2,16,21] and our results suggest that downregulation of Pax3 is prerequisite in eliciting ectopic Pax7 expression, and further that this may provide evolutionary potential that is associated with genuine function even if this function is not essential or has yet been ascertained [55]. Thus, rather than genetic redundancy, Pax7 is acting in a functional genetic compensation manner in *Pax3^neo/Δ5^* hypomorphs, and reduced Pax3 repression empowers compensatory ectopic Pax7 expression. Additionally, the fact that Pax3 and Pax7 are multifunctional proteins with bipartite DNA binding domains and that both can form homo- and hetero-dimers on DNA [6] enables the observed Pax7 compensatory ectopic upregulation and functional genetic compensation capability.

In summary, using unique *Pax3^neo/Δ5^* hypomorphs, we titrated the minimally required Pax3 level to be 10% and discovered exacerbated defects in neural tube closure, neural crest emigration, outflow tract septation, and craniofacial and musculature morphogenesis. We also demonstrated that the cardiac neural crest cell lineage is one of the most resistant *Pax3*-expressing cell lineages to Pax3 reduction. Using compound mutant models, we further documented that cardiac neural crest resistance is attributed to a functional genetic compensation role mediated via ectopic and upregulated Pax7 expression within the *Pax3^neo/Δ5^* dorsal-most neural tube. We also demonstrated that either systemic or lineage-restricted loss of Pax7 results in unanticipated severe craniofacial defects in *Pax3^neo/Δ5^* hypomorphs. These findings implicate that reduced levels of PAX3 and PAX7 expression due to *PAX3* and/or *PAX7* polymorphisms may underlie *in utero* outflow tract or craniofacial malformation in patients.

## Figures and Tables

**Table 1:** Listing PCR primers with insert sizes, specific annealing (AT) temperatures and PCR cycle number. # stands for number.

Gene	Forward Primer	Reverse Primer	Size (bp)	AT (oC)	Cycle #
*Pax1*	GACTGGGCGGGTGTGAACCG	GTGCATCCGTGGCCTTGCCT	361	58	33
*Pax2*	CGGATGGGGCAGGGACAGGA	CTGCCCAGCTCAGGGTTGGC	430	68	36
*Pax3 5*′	GTCTCGCCTTCACCTGGATA	GTTGTCACCTGCTTGGGTTT	379	58	32
*Pax3 3*′	GATGGAGGAAACAAGCTGGA	GATGGAGGCACAAAGCTGTC	304	58	32
*Pax3 exon5-6*	TTACCGCTGAAGAGGAAG	GTGTACAGTGCTCGGAGGAAG	383	58	32
*Pax4*	GGCTCCCAGTGTGTCCTCTA	CCCATTTCAGCTTCTCTTGC	350	64	36
*Pax5*	CAGGGACCGGCTGTTGGCAG	GGACTGTGGGCCTGGAACGC	553	58	31
*Pax6*	CCGCAGCACTCGAGCACCAA	GAGCTGGTTGGCAGCCTCCG	471	58	33
*Pax7*	AAGTGTCCACCCCTCTTGG	TGATTCTGAGCACTCGGCTA	399	64	36
*Pax8*	AGGCCTATGCCTCCCCCAGC	GGAAGCGCCAGGCCTCACTG	537	66	33
*Pax9*	GGGCCGGGTTGGTGTACTGC	GCGGGCTGGTGCTGCTTGTA	474	66	31
*GAPDH*	ACCACAGTCCATGCCATCAC	TCCACCACCCTGTTGCTGTA	452	58	25

## Supplementary Materials

The following supporting information can be downloaded at: https://www.mdpi.com/article/10.3390/jdb10020019/s1, Figure S1: Impaired splicing results in reduced Pax3 mRNA expression in Pax3Δ5/neo hypomorphic mutants; Figure S2: Pax7Cre knockin recapitulates endogenous Pax7 expression.
