# Supplementary material for: Pax3 Hypomorphs Reveal Hidden Pax7 Functional Genetic Compensation in Utero
Source: J Dev Biol. 2022 May 17;10(2):19. doi: 10.3390/jdb10020019 (PMC9149870; doi:10.3390/jdb10020019)
Supplement: Supplementary file 1 [file jdb-10-00019-s001.zip › jdb-1702363-supplementary.pdf]

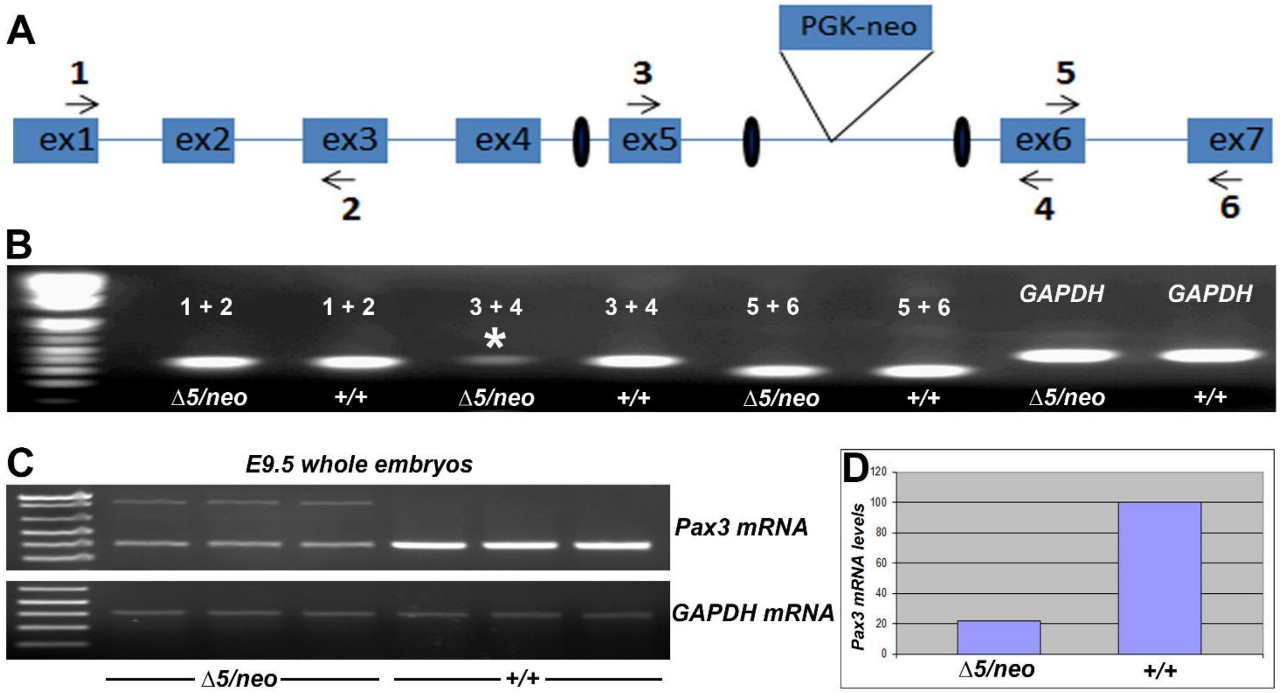

Supplemental Figure S1. Impaired splicing results in reduced *Pax3* mRNA expression in *Pax3*$^{\Delta 5/neo}$ hypomorphic mutants. (A) Schematic of *Pax3*^{neo} allele. Numbered arrows indicate the location of three sets of forward (on top) and reverse (on bottom) *Pax3* PCR primer pairs. *Exon5* and *PGK-neo* cassette are each flanked by loxP sites. **(B)** RT-PCR analysis of *Pax3* and *GAPDH* mRNA expression levels from E9.5 pooled whole *Pax3*$^{\Delta 5/neo}$ and wildtype (+/+) embryo lysates. The strategy of primer combination (1+2) and (5+6) amplifies wildtype, *Neo* and *Δ5* mutant allele *Pax3* mRNA at 5′ (1+2) and 3′ (5+6) regions, revealed that *Pax3*^{neo} and wildtype mRNA levels are similar. However, the primer combination (3+4) amplifies only wildtype *Pax3* mRNA (as PGK-neo band is too large to amplify within PCR cycle time), and this reveals that in *Pax3*$^{\Delta 5/neo}$ mutants there is a >80% reduction (indicated by *) in *Pax3* mRNA relative to wildtype levels. *GAPDH* was used as a loading control. **(C)** RT-PCR detection of *Pax3* in triplicate E9.5 *Pax3*$^{\Delta 5/neo}$ and wildtype embryos. Note when *Pax3* mRNA was amplified using primer combination (3+4) with prolonged extension times, this enables detection of the abnormally spliced amplicon (top band 850bp) in *Pax3*$^{\Delta 5/neo}$ mutants but not controls. This indicates exon5-6 splicing of mRNA transcribed from *Pax3*^{neo} allele is impaired due to presence of PGK-neo cassette. **(D)** *Pax3* mRNA levels was determined by densitometry and normalized to *GAPDH*. Relative to 100% expression in wildtype, there is only ~20% normal *Pax3* within the E9.5 *Pax3*$^{\Delta 5/neo}$ mutants.

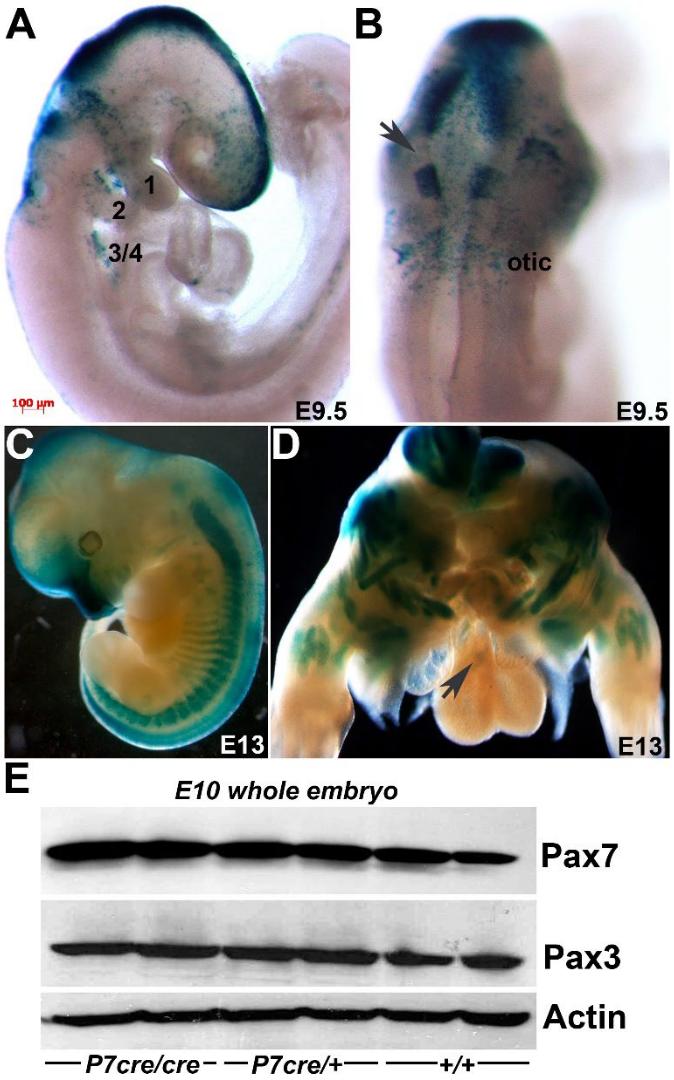

Supplemental Figure S2. ***Pax7****^{Cre}* **knockin recapitulates endogenous** ***Pax7*** **expression. (A,B)** Right lateral (A) and dorsal (B) view of *Pax7*^{Cre}/*R26r*^{LacZ} X-Gal stained E9.5 embryo, illustrating only robust reporter expression within cranial neural tube and craniofacial region but not more caudal regions. Note *Pax7*-marked neural crest emigration streams towards the pharyngeal arches (1-4), and the regional localization of *Pax7*^{Cre} within the hindbrain and absence from rhombomeres 3 (arrow in B) and 5. **(C,D)** Left lateral view at whole E13 X-Gal stained embryo and same embryo with head removed. Arrow in (D) points to a small population of *lacZ* cells that have colonized the normal outflow of the heart. **(E)** Western analysis of Pax7, Pax3 and βActin levels in duplicate *Pax7*$^{Cre/Cre}$, *Pax7*$^{Cre/+}$ and wildtype (+/+) E10 whole embryo lysates. Note that all three genotypes express similar levels of Pax7 and Pax3, indicating that the knockin of *Cre* cDNA at the *Pax7* 3'UTR does not affect endogenous Pax7 not Pax3 expression levels. Scale A,B, 100µm.
